# From propaganda to neoliberal consumer culture: the cultural logics of physical activity promotion in Maoist and urban China

**DOI:** 10.3389/fpubh.2025.1687450

**Published:** 2025-09-23

**Authors:** Pengyu Zhu, Zhihui Pang, Yuheng Yin, Zhixiang Wen, Yang Zhang

**Affiliations:** ^1^College of Physical Education, Hunan Normal University, Changsha, China; ^2^College of Politics and Administration, Tianjin Normal University, Tianjin, China; ^3^Independent Researcher, Windermere, FL, United States

**Keywords:** exercise, identity, health behavior, health promotion, social media, symbolic capital

## 1 Introduction

As global societies confront rising rates of obesity, inactivity, and aging, the promotion of physical activity has become an urgent public health priority (1). Yet in China, physical activity is not merely a biomedical or behavioral concern—it is a deeply cultural practice, shaped by shifting logics of governance, identity, and aspiration.

This opinion article argues that physical activity in China has evolved through three overlapping cultural logics. First, during the Maoist era, movement was a tool of ideological discipline, symbolizing loyalty and collectivism through mass drills and political performance. Second, the post-reform era saw the rise of state-led modernization, where the National Fitness Program framed exercise as both a civic duty and a vehicle for national development. Today, a third logic dominates: that of consumer capitalism culture, where fitness is increasingly driven by market forces, aesthetic aspiration, and lifestyle branding. This shift reframes physical activity as symbolic capital: pursued not just for health or discipline, but for identity, experience, and social distinction. Across domains ranging from marathons and outdoor recreation to digital fitness and winter sports, participation increasingly reflects aspirations shaped by consumer media and cultural taste in urban China.

By tracing this transition—from propaganda to consumerism—we call for a rethinking of how physical activity is promoted and understood. If movement is increasingly about meaning, distinction, and aspiration, then public health strategies must go beyond biomedical logic and engage with the deeper cultural infrastructures that shape why, how, and for whom fitness matters—not only in China but globally.

## 2 Theoretical foundations: culture, capital, and the shifting meaning of movement

Physical activity is often framed in public health as a rational behavior: individuals are encouraged to move more to prevent disease, reduce risk, or increase lifespan. Yet this view obscures the cultural conditions under which movement acquires meaning. Fitness is never purely about physiology—it is a socially constructed, symbolically encoded practice embedded in values, aesthetics, and social order.

This article adopts a cultural perspective, drawing on the work of Zygmunt Bauman and Pierre Bourdieu to conceptualize how the meaning of physical activity in China has shifted across historical phases. In Bauman's “liquid modernity” (2), individuals are no longer tethered to fixed social roles or collective ideologies. Instead, they must continually curate their own identities through flexible, often market-based choices. Movement, in this context, becomes part of the neoliberal self—a form of self-management that reflects personal responsibility, adaptability, and aesthetic coherence.

In parallel, Bourdieu's theory of cultural capital offers a lens to understand how fitness practices serve as tools of social distinction (3). The type of exercise one does, the gear one wears, and the bodies one aspires toward all operate as cultural signals (4). In today's China, the rise of boutique outdoor brands, marathon culture, winter sports fashion, and outdoor lifestyle branding reflects more than an interest in health—it reflects an emerging regime of aesthetic and class-based stratification. Through fitness, individuals accumulate symbolic capital, signaling upward mobility and lifestyle fluency (5).

Together, these perspectives suggest that physical activity is not merely shaped by policy or health messaging, but by deeper shifts in cultural logic. From the collectivist choreography of Maoist drills to the aspirational consumerism of digital fitness and winter sports, movement practices mirror the governing ideologies of their time. Public health cannot be separated from these dynamics; to be effective, it must confront them directly.

## 3 Historical review: from collectivism to marketized self-improvement (1950s−2010s)

The cultural meaning of physical activity in China has shifted dramatically over the past seven decades, reflecting transformations in ideology, governance, and individual subjectivity. During the Maoist period, movement was a political act. Public exercise served as both physical training and ideological choreography—mass calisthenics, military-style drills, and revolutionary operas (6) aimed to cultivate collectivist discipline and national strength (7). Fitness symbolized loyalty, modernity, and alignment with state ideology. Even traditional forms like Taijiquan were recoded to serve revolutionary narratives or sidelined altogether (8). Movement was not about health, but about national cohesion through embodied propaganda (9).

With the launch of Deng Xiaoping's reform and opening-up policies in the late 1970s, the logic of fitness began to shift. Market liberalization introduced Western beauty standards, personal health awareness, and commercial gym culture (10). Grassroots mass sports and professional competitions emerged in the 1980s and 1990s—not as collective rituals, but as tools of individual enhancement (11). The National Fitness Program, launched in 1995, signaled an institutional response to rising health concerns, but also reflected an ideological pivot: from collectivist duty to self-responsibility. This era marked the convergence of state guidance and consumer logic. Physical activity was still promoted by the state but increasingly practiced through personal choice, privatized infrastructure, and lifestyle aspiration. The body became a site of self-improvement rather than ideological loyalty—a shift that prefigured the neoliberal logics of the 21st century. Yet even in this transition, state influence remained strong. National fitness campaigns, school physical education requirements, and public park infrastructure coexisted with the expansion of commercial gyms and beauty-centered fitness trends. This dual structure illustrates the hybrid nature of China's modernization: a blending of civic nationalism with market-driven individualization.

By the early 2000s, physical activity in urban China had become a symbolic battleground—where collectivist values, state modernization goals, and emerging consumer lifestyles collided. This layered terrain set the stage for the contemporary phase: one in which urban fitness practices are increasingly shaped by consumer aspiration, lifestyle signaling, and health discourse, mediated through digital platforms and global branding.

## 4 Cultural drivers of fitness: how neoliberal consumer culture is shaping urban physical activity in China

In today's urban China, fitness is no longer just a public health initiative or state mandate—it is a culturally curated activity, governed by consumer aspiration, social distinction, and digital visibility. Across diverse domains of physical activity, neoliberal consumer culture has emerged as the dominant force shaping how, why, and for whom people move. This shift is not merely about privatization or market expansion; it reflects a deeper transformation in the symbolic and aesthetic meanings of fitness.

The mass marathon boom offers the clearest example of this transition. Once exclusive to elite athletes, marathons have exploded across urban China as mass-participation spectacles that blend personal aspiration, civic pride, and commercial branding. From tier-one cities like Beijing and Shanghai to emerging hubs like Xiamen and Chengdu, marathon slots sell out within minutes. Participation is driven by multiple overlapping forces: health consciousness, symbolic self-achievement (documented online through medals, race bibs, and finish-line photographs), and middle-class lifestyle signaling. As runners post-GPS routes, branded gear, and finish times on Xiaohongshu or WeChat Moments, the act of running becomes a form of personal narrative construction—a demonstration of discipline, upward mobility, and bodily control (12–14). These events are also co-produced by state and market actors (15, 16). Municipal governments support them to promote Healthy China 2030, attract tourism, and signal modern urban identity. Global brands—Nike, Anta, Li-Ning—sponsor and shape the visual culture of these races. In this sense, marathons reflect a soft neoliberal governance model, in which the state fosters individualized health behavior within a consumer-driven and media-saturated environment.

A related transformation can be seen in outdoor sports culture, where fashion, fitness, and identity now intersect. Brands like Arc'teryx and Toread have evolved into urban status symbols, worn less for backcountry function and more for social signaling. Documenting hikes, trail runs, or weekend camping trips on Xiaohongshu has become a digital ritual of identity performance, particularly among urban youth. Participation in outdoor fitness is no longer about survival or nature immersion—it is a curated lifestyle aesthetic (17), deeply informed by global fashion and local platform culture.

Meanwhile, digital platforms and fitness apps like Keep, Douyin, and Bilibili are transforming how fitness is experienced and consumed. Workouts are personalized, gamified, and aestheticized. Algorithms prioritize content that conforms to specific body ideals—leanness, tone, flexibility—thus shaping fitness aspiration through visual norms (18). App-based programs offer not only training plans but fitness personas, each with their own branded routines, language, and gear. Bauman's “liquid modernity” resonates here: in a world without fixed identity roles, the body becomes a flexible, self-managed project requiring constant attention, alignment, and presentation.

Finally, the rapid expansion of modernized winter sports, especially after the 2022 Beijing Olympics, reflects how state strategy and consumer culture now operate in tandem. With the slogan “300 million people on snow and ice,” China launched a nationwide campaign to popularize skiing and snowboarding (19). Yet the infrastructure—indoor ski resorts like L+Snow, luxury winter gear, and influencer-driven aesthetics—signals a new kind of aspirational consumption. Skiing becomes less a national movement and more a premium urban experience, accessible through wealth, style, and social visibility.

Taken together, these examples demonstrate that urban fitness in China is now culturally governed by neoliberal consumer logic. Movement practices are filtered through brand meaning, aesthetic aspiration, digital performance, and individualized self-construction. Public health strategies, while still active, increasingly compete with—or are absorbed by—this symbolic economy. To understand contemporary physical activity, we must therefore engage not just with policies or environments, but with the cultural infrastructures that shape why movement matters, what it represents, and who gets to participate in its most desirable forms.

## 5 Fitness beyond behavior: cultural strategy for public health in the consumer age

**Figure 1:**
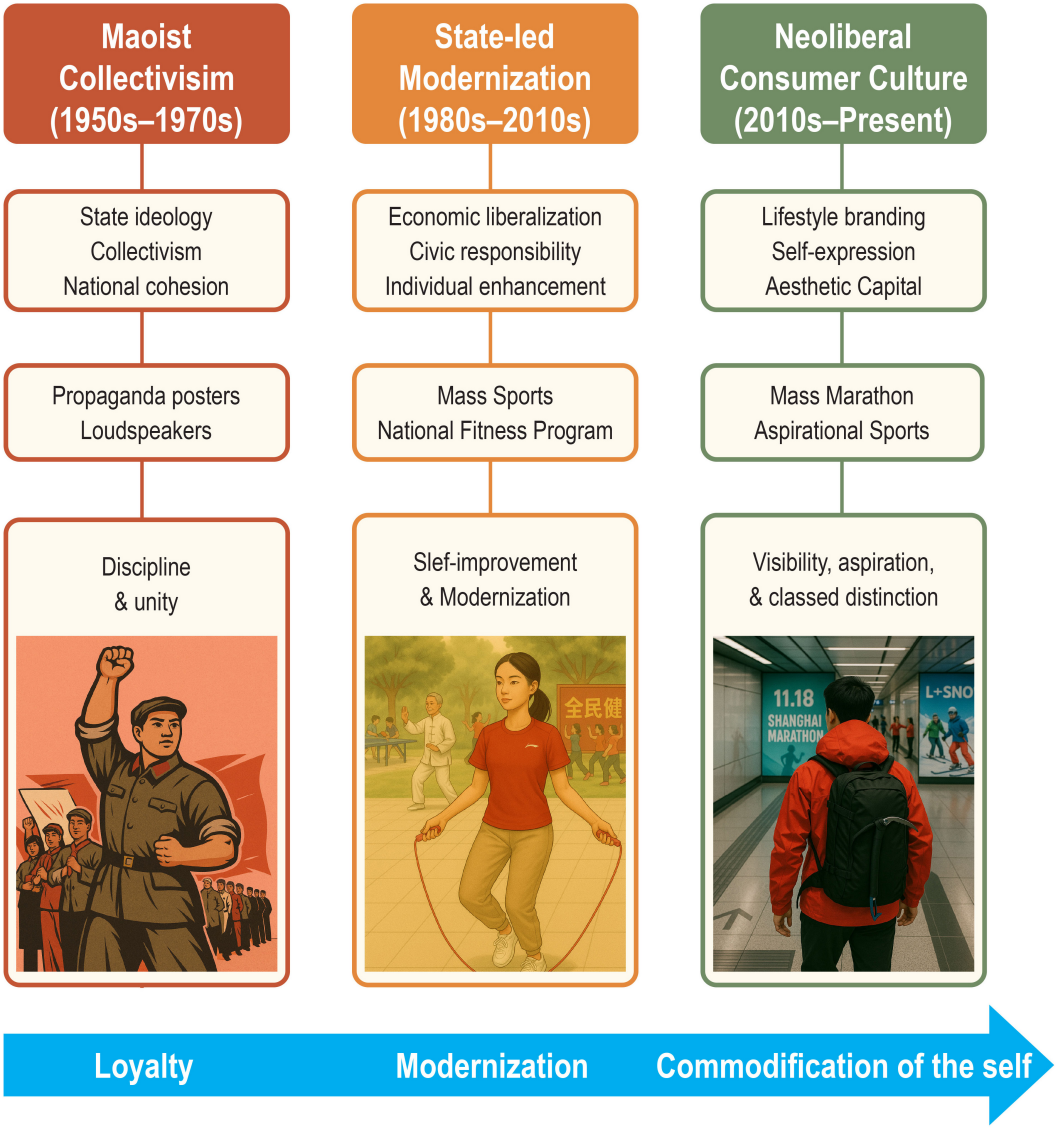
Cultural evolution of physical activity in China (1950s–present).

Figure 1 illustrates the cultural evolution, tracing the dominant logic, practices, and meanings of physical activity across China's recent history. If public health promotion is to remain relevant in the 21st century, it must adapt to the cultural logics that govern how people understand and experience movement. In urban China, physical activity is no longer shaped primarily by policy, infrastructure, or health education; the latest logic reflects the commodification of the self, where fitness becomes not just health behavior but symbolic performance—curated, branded, and algorithmically reinforced. This shift requires a fundamental rethinking of health promotion strategy.

First, policymakers must engage cultural producers as collaborators, not just conduits. Influencers, platforms, and lifestyle brands already shape the visual and symbolic landscape of fitness. Rather than regulating from the outside, public health agencies should co-create with these actors to elevate inclusive, evidence-based content. Second, public investment must support not only access but meaning. Even well-designed parks, gyms, and apps may be underused if they do not resonate with people's identities or aspirations. Health promotion should center movement as an expressive, identity-driven act—not just a utilitarian one. Third, cultural inclusion must go beyond demographic targeting to symbolic representation. Marginalized bodies—older adults, rural residents, disabled individuals—must be seen and celebrated in aspirational fitness culture. This means subsidizing and platforming diverse forms of movement, including square dancing, martial arts, or rural walking clubs, and ensuring they are not symbolically excluded from what fitness “looks like.”

China's marathon boom, winter sports market, and fitness app ecosystems illustrate how cultural infrastructures—not just physical ones—can scale health engagement. Other countries should study how meaning and aspiration drive participation, and build promotion models accordingly.

## 6 Discussion

This article has traced the evolving role of culture in shaping physical activity, from Maoist collectivism, state-led modernization, to the era of neoliberal consumer culture. Each phase reflects distinct symbolic meanings of movement—loyalty, discipline, self-improvement, and now self-branding and lifestyle aspiration. Understanding this evolution reveals why biomedical and behavioral models alone are insufficient. Fitness is not simply a matter of access or awareness; it is a cultural practice governed by shifting norms, aesthetics, and media ecologies. The China case is instructive for public health globally—not because it can be copied wholesale, but because it shows how culture actively produces the conditions of participation.

At the same time, this analysis is not without limits. It focuses primarily on urban, middle-class experiences and does not fully capture fitness cultures in rural areas or among older and disabled populations. These gaps should be addressed in future work.

Ultimately, if public health is to inspire real movement, it must engage with what movement means. People do not simply move to avoid disease—they move to perform identity, express belonging, and construct a future self. Health promotion must recognize that fitness is not only physical—it is symbolic, aspirational, and culturally governed.
